# P-1453. Disparities in PrEP utilization

**DOI:** 10.1093/ofid/ofae631.1625

**Published:** 2025-01-29

**Authors:** Ayesha Soomro

**Affiliations:** University of Cincinnati/Mercy South St. Louis, St. louis, Missouri

## Abstract

**Background:**

In October 2023, the CDC released data regarding PrEP uptake in the United States. The data indicated that in 2022, for the first time, more than one-third of individuals who could benefit from PrEP had been prescribed it. While this advancement is certainly significant, as PrEP coverage is one of the cornerstones in ending the HIV epidemic, the report also highlighted substantial disparities.

According to the report, while 94% of White individuals who could benefit from PrEP had been prescribed it, only 13% of Black and 24% of Hispanic/Latino individuals who could benefit had received prescriptions for PrEP. Another notable disparity was observed regarding sex at birth, with 40% of males who could benefit from PrEP being prescribed it, compared to only 15% of females.

**Figure:**
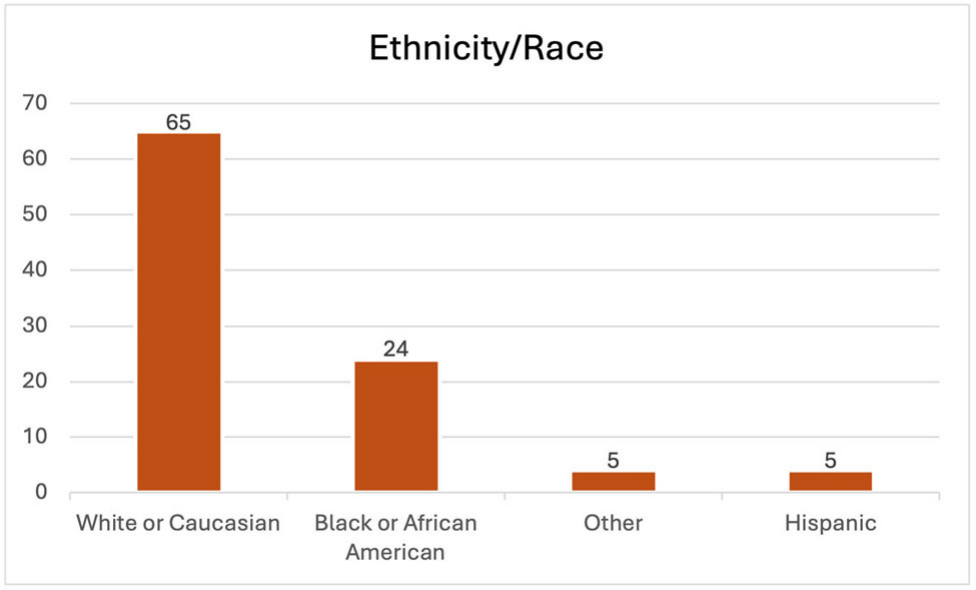

**Methods:**

We aimed to determine if such a magnitude of disparity existed within our patient population receiving care at the infectious disease clinic. We identified 99 patients who had been prescribed PrEP at the ID clinic. These individuals were categorized based on their self-identified race/ethnicity and sex assigned at birth.

**Figure:**
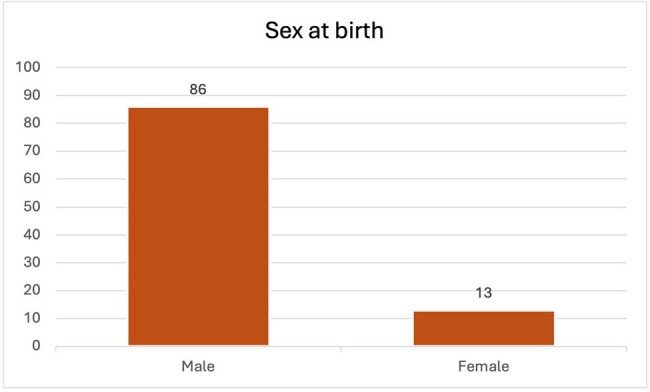

**Results:**

Of the 99 individuals prescribed PrEP, 86 were males, while only 13 were females. Regarding race/ethnicity, there were 65 White or Caucasian patients, 24 Black or African American patients, 5 Hispanic or Latino patients, and 5 others.

**Conclusion:**

Compared to national data published by the CDC, our data shows similar disparities. While the PrEP-to-need ratio would be a better indicator of the true disparity, our data still highlights the trend in racial and sex-based differences among users of PrEP prescriptions.

**Disclosures:**

**All Authors**: No reported disclosures

